# STATISTICAL APPROACH FOR HUMAN ELECTROMAGNETIC EXPOSURE ASSESSMENT IN FUTURE WIRELESS ATTO-CELL NETWORKS

**DOI:** 10.1093/rpd/ncy120

**Published:** 2018-07-30

**Authors:** Sergei Shikhantsov, Arno Thielens, Günter Vermeeren, Piet Demeester, Luc Martens, Guy Torfs, Wout Joseph

**Affiliations:** 1 Department of Information Technology, Ghent University/IMEC, Ghent, Belgium; 2 University of California Berkeley, Department of Electrical Engineering and Computer Sciences, Berkeley Wireless Research Center, Berkeley, CA, USA

## Abstract

In this article, we study human electromagnetic exposure to the radiation of an ultra dense network of nodes integrated in a floor denoted as ATTO-cell floor, or ATTO-floor. ATTO-cells are a prospective 5 G wireless networking technology, in which humans are exposed by several interfering sources. To numerically estimate this exposure we propose a statistical approach based on a set of finite difference time domain simulations. It accounts for variations of antenna phases and makes use of a large number of exposure evaluations, based on a relatively low number of required simulations. The exposure was expressed in peak-spatial 10-g SAR average (psSAR_10g_). The results show an average exposure level of ~4.9 mW/kg and reaching 7.6 mW/kg in 5% of cases. The maximum psSAR_10g_ value found in the studied numerical setup equals around 21.2 mW/kg. Influence of the simulated ATTO-floor size on the resulting exposure was examined. All obtained exposure levels are far below 4 W/kg ICNIRP basic restriction for general public in limbs (and 20 W/kg basic restriction for occupational exposure), which makes ATTO-floor a potential low-exposure 5 G candidate.

## INTRODUCTION

**Figure 1.**
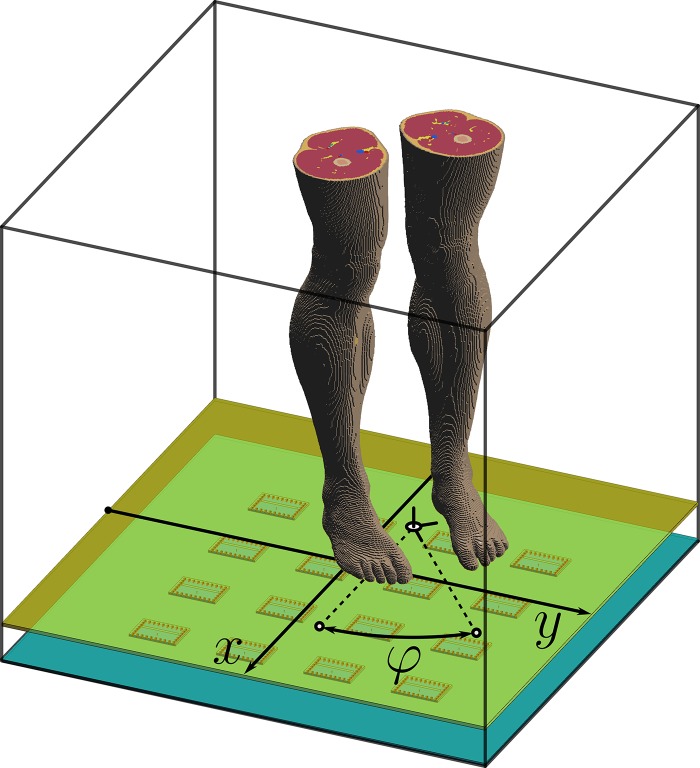
Simulation domain. The 16 patch-antennas and voxeled part of the phantom included into the domain are shown. *x* and *y* are Cartesian coordinates the phantom’s pivot point in horizontal plane and angle φ defines the phantom’s rotation normal to the plane. Black wireframe box shows the boundaries of the domain.

The ATTO-floor is a new concept for ultra-high capacity wireless networking, designed to provide wireless access to robots that can freely move around the floor surface. ATTO-cells are integrated into the floor and cover its entire area. According to the current design^(1)^ (Figure 1) an ATTO-cell has dimensions of 15-by-15 cm^2^ and an antenna is supplied with a maximum power of 1 mW. It operates at a center frequency of 3.5 GHz. Possible applications of the ATTO technology include industrial warehouses or factories of the future, where multitudes of mobile robots and human workers operate simultaneously. Robots, being equipped with an antenna featuring downward-pointing pattern, are the target users. Due to the provisioned fast handover system, at any time instance a robot is only connected to the closest antenna, thus, it is unlikely for humans to be exposed by the ATTO-floor directly. In other words, most of the time humans will be exposed to the scattered fields of antennas serving surrounding robots.

Exposure from a single ATTO-cell was studied both numerically and experimentally in Ref.^(2)^. Peak spatial specific absorption rate averaged over a 10g cube (psSAR_10g_) was found to be lower than 2.8 mW/kg, which is far below International Commission on Non-Ionizing Radiation Protection (ICNIRP) guidelines for the general public in limbs (4 W/kg). Though the power radiated by the ATTO-cell is not enough to violate the ICNIRP guidelines, the ATTO-floor network represents a valuable study-case of exposure to phased antenna-arrays. Moreover, appropriate scaled obtained exposure levels remain valid for an arbitrary antenna radiated power.

In this article, we address, for the first time, the worst-case exposure scenario for an ATTO-floor network: a human standing on the entire ATTO-floor network with all antennas radiating simultaneously and constantly. To assert the highest exposure we need to account for the fields induced by the antennas in proximity of the studied subject. As it will be shown further, the number of antennas that give significant contribution to the total exposure is sufficiently small. Another novelty of this article is the development of a statistical approach for exposure estimation in a system with multiple interfering nodes. Applying this approach, we evaluated the psSAR_10g_ that could hypothetically be produced by ATTO networks under very conservative assumptions.

In a multi-antenna system the powers and the relative phases with which the antennas are supplied define the resulting electromagnetic (EM)-field distribution^(3, 4)^. This affects the power dissipated in the regions occupied by human body tissues. To find such a combination of powers and phases that yields the highest exposure means finding the worst-case exposure in a given scenario. A method which addresses this problem in the case of the exposure to multi-coil MRI-scanners is known^(5, 6)^. It can be shown that, if the total power shared by all the antennas is limited, than the problem is equivalent to finding the largest eigenvalue of a matrix. However, if the maximum power is limited per antenna, a general optimization method is needed^(7)^. In the following sections we propose a statistical approach that not only allows to find the worst-case, but also gives an estimate of the average exposure and field distribution over the ATTO-floor.

## MATERIALS AND METHODS

In the first part of this section the numerical setup is presented. In the second part we give an overview of simulations that were conducted. In the third part the post-processing methods are provided and the method of exposure assessment is explained.

### Numerical setup

For EM simulations we used the finite-difference time-domain (FDTD) method implemented in Sim4Life v3.2 (ZMT, Zürich, Switzerland). The simulation domain is depicted in Figure 1. We used the Virtual Population v.3.1 posable heterogeneous Duke phantom^(8)^, which represents an average adult male human (height = 1.77 m, mass = 70.2 kg, BMI = 22.4 kg/m^2^). Its feet were rotated by 10° in the sagittal plane to be parallel to the floor, as it usually is in a normal standing posture. The shortest distance between the feet and the surface of the floor is 10 mm, which is aimed at representing the height of a shoe sole. The floor surface is a sheet of 6 mm Acrylic glass. A 4-by-4 array of equidistantly separated patch antennas is placed on a plastic $\left(\sigma =5\cdot 10^{-4}\;S/m,\;\varepsilon_{r}=2.25\right)$ substrate 58 mm below the floor to form 16 ATTO-cells. The sensitivity of the exposure values to the antenna array size is studied quantitatively in the next section and the choice of 4-by-4 array is justified.

In order to optimize computational resources, only the legs of the phantom were included into the simulation domain. This change has a negligible effect on the field distribution inside the phantom due to its fast decrease in amplitude with distance from the floor (more than 50 dB at 1 m height, see Ref.^(2)^). The domain boundary box dimensions were set to be 750-by-750-by-1200 mm^3^ and absorbing boundary conditions with perfectly matched layer (PML) were applied. With the maximum of 1.2 mm discretization resolution in lossy regions, it resulted in ~150 million voxels in total.

In the given setup, the phantom penetrates the near-field region of at least some of the antennas in the array $\left(2L^{2}/\lambda \approx 220\;\mathrm{mm}\right)$ and the exposure is highly affected by its location with respect to the array. To study this effect we allow phantom translation in the horizontal plane (parallel to the floor) and rotation around an axis orthogonal to the floor. Such transformation can be defined by three scalar parameters: two Cartesian coordinates *x*, *y* and the angle of rotation φ. Any fixed set of {x,y,φ} we will further refer to as configuration, see Figure 1. Exposure variation due to the phantom’s movement in the direction perpendicular to the floor was covered in Ref.^(2)^, and is not considered in the current study.

### Simulations

**Figure 2.**
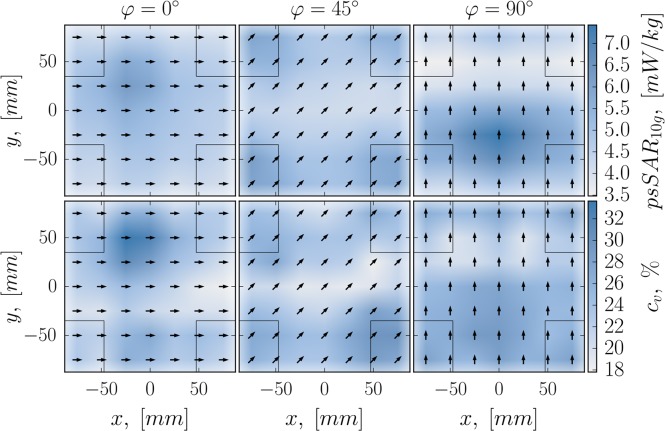
Average value (top) and relative standard deviation $c_{v}$ (bottom) of exposure samples distribution for all configurations. Arrows denote the rotation of the phantom. The outlines of the antennas are shown with thin black lines. The color-bars on the right show SAR_10g_ in mW/kg (top) and $c_{v}$ in % (bottom).

We assumed that all positions and orientations of a human on the floor have equal probability. Utilizing the periodical structure of the antenna array, we restricted translations of the phantom to a central rectangle of size 150-by-150 mm^2^, which matches the size of one ATTO-cell. The rectangle is covered with a 7-by-7 rectangular grid of nodes which are equidistantly separated, see Figure 2. Taking into account reflection symmetries of individual patch antennas relative to *x* and *y* axis and matching symmetries of the antenna array structure, for each node of this translational grid we considered three angles of phantom’s rotation: 0°, 45° and 90°. In total we obtain 147 configurations in which the phantom is translated to one of the nodes on the grid and rotated to one of the angles.

For every configuration we performed a multi-port FDTD simulation. A multi-port simulation consisted of 16 single-port simulations. In each of them only a single antenna is excited with a 3.5 GHz sinusoidal signal of normalized input power. After the simulation reached a stationary state, electric fields in a sub-region that encloses only the phantom’s feet are saved for post-processing.

### Post-processing

The post-processing is done in several steps.

First, we assume that antennas driven with maximum power lead to the highest exposure. This allows to normalize the field distributions obtained from simulations to the radiated power of 1 mW.

Second, we independently sample 16 numbers from a uniform random distribution in [0, 2π). These are set as phases for antennas in a multi-port simulation. By doing so we assume that the phases of antenna signals are uncorrelated.

Third, we calculate a field distribution inside the domain with amplitudes and phases of all 16 antennas set. Using standardized numerical routines (IEC/IEEE P62704-1) we obtain the psSAR_10g_ value, which is further referred to as an exposure sample.

Fourth, we generated 10^3^ exposure samples for each configuration. This yields 147 × 10^3^ exposure samples in total, which cover variations of phantom positions on the floor and antennas relative phases. This sample set allows to estimate the statistical properties of exposure in the given EM-environment.

The procedure of exposure samples generation can also be viewed as a Monte-Carlo random point method for finding a global minimum of a function. The numerical error of this method decreases with the number of samples *N* as $1/\sqrt{N}$^(9)^. To further decrease the numerical error, we use phases of the highest exposure sample as a starting point for an optimization. The complete numerical procedure is integrated into the Sim4Life scripting environment, which allows to utilize its internal algorithms for psSAR_10g_ evaluation at every iteration. The resulting solution is deemed to approach closely the upper bound of the SAR_10g_ in a given configuration.

Finally, we examined the effect of human body morphology on peak SAR in the worst-case configuration. For it we performed additional FDTD simulations with three heterogeneous Virtual Family V1.0 phantoms^(10)^, Ella, an adult woman, Billie, an 11-year-old girl and Thelonious, a 6-year-old boy. Each of these phantoms was simulated in the configuration, in which the worst-case exposure was found for the Duke phantom (adult man). Using this configuration aims at providing an approximation for the worst-case exposure avoiding a computationally expensive process of determining it more accurately, as it was done previously for Duke. Each phantomâ€™s feet were rotated to be parallel to the ground and the simulation domain dimensions were preserved. Such approach allows detecting phantom-related factors influencing the exposure (e.g. size of the feet), which magnitude is greater than the variation of the exposure across the ATTO-floor.

## RESULTS

Figure 2 shows spatial distribution of exposure for the simulated configurations. The color of a square corresponds to the mean value (top row) and relative standard deviation $c_{v}$ expressed in % (bottom row) of 1000 exposure samples. Its coordinates in the *xy*-plane match the coordinates of the phantom’s pivot point in a configuration. Columns represent the angle of the phantom’s rotation. These exposure maps give a high-level summary of exposure variation inside the ATTO-cell.

In general, the highest mean psSAR_10g_ is observed when the toes of one of the feet are placed directly above the feed-point of an antenna, e.g. {0, −25 mm, 90°}, see Figure 2. Higher maximum mean values are observed when the feet are perpendicular to the polarization direction of the antennas (90°), whereas their parallel mutual placement (0°) results in lower mean SAR_10g_ values, which are more evenly distributed over the *xy* plane. One possible reason for that is the occurrence of a resonance in the toes in the former case^(11)^.

**Figure 3.**
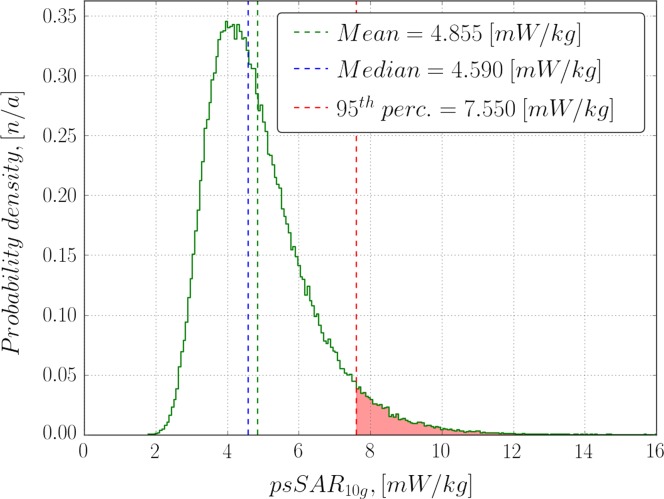
Histogram of all generated exposure samples pooled together. Emitted power per antenna is 1 and 15 mW in total. Mean and median of the distribution are shown in dashed blue and green lines, respectively, and values exceeding the 95th percentile are shown in red.

To obtain general characteristics of the ATTO-floor exposure, all samples from all configurations were plotted as a histogram in Figure 3. It is a bell-shaped skewed distribution. Its nonparametric skew, defined as S=(μ−ν)/σ, where *μ* is its arithmetic mean, *ν* is the median and *σ* is the standard deviation, equals ~0.19. Its arithmetic mean can be interpreted as an average exposure of ATTO-floor and equals 4.9 mW/kg. This is almost twice the upper limit found in Ref.^(2)^ for a single ATTO-cell but still three orders of magnitude lower than ICNIRP general public guidelines (4 W/kg). 95th percentile indicates the level of exposure that is exceeded with 5% chance and was found to be around 7.6 mW/kg.

**Figure 4.**
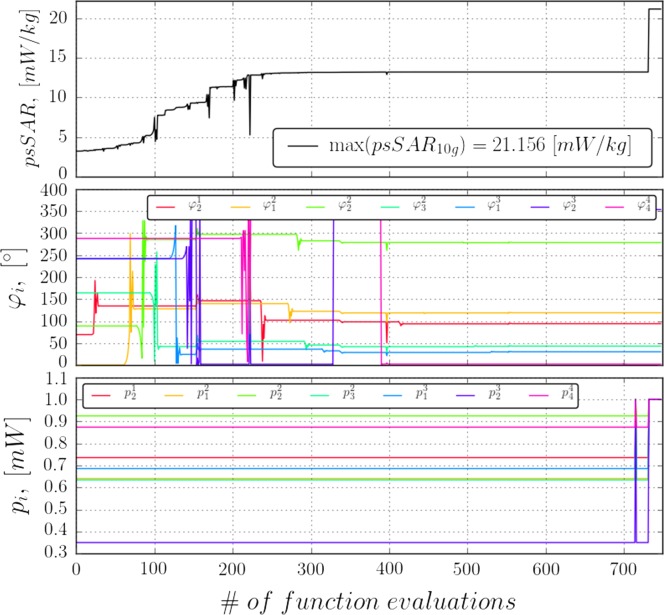
The evolution of optimization procedure with coupled modified Hybrid Powell and L-BFGS-B methods implemented in SciPy Python library. Changes of psSAR_10g_ are shown (top) along with the relative phases of selected antennas (middle) and their powers (bottom).

To establish the upper bound on the exposure of the ATTO-floor we perform an optimization procedure. We use the configuration yielding the highest sample mean psSAR_10g_ (7.4 mW/kg) and, at the same time, contains a sample with highest exposure value (14.9 mW/kg). This configuration is defined by the set $\left\{0\;\mathrm{mm},-25\;\mathrm{mm},90^{\circ }\right\}$. In this case, the toes of the phantom are located directly above two central antenna tiles. Phases and powers of all 16 antennas act as minimization parameters of the objective function $-\;\log\left({\mathrm{psSAR}}_{10g}\right)$, therefore maximizing psSAR_10g_. Two optimization algorithms are coupled: first phases are optimized using Hybrid Powell method^(12)^, then powers of antennas bounded between 0 and 1 mW are optimized with the L-BFGS-B algorithm^(13)^. The procedure is performed 100 times; each time the parameters to initialize the optimization are independently sampled from the uniform random distribution: in [0,2π] for phases (in radians) and in [0,1] for powers (in mW). An example of the evolution of a successful optimization procedure is depicted in Figure 4.

**Figure 5.**
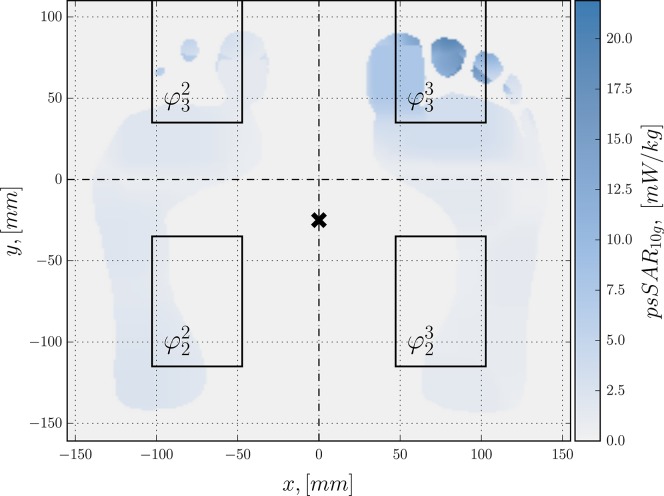
Top view of the worst-case psSAR_10g_ distribution in the horizontal slice coincident with the peak location. Antenna outlines are shown as black rectangles with their indices indicated at the lower left corner. A black cross depicts the phantom’s pivot point.

The antennas were numbered using two indices: the lower index indicates antenna number along the *y*-axis, the upper index indicates antenna number along the *x*-axis. Phase $\varphi_{3}^{3}$ has the highest impact on the psSAR_10g_, with corresponding antenna being located directly under the peak-SAR cube (Figure 5). Phase $\varphi_{3}^{3}$ was set as a reference and phases of antennas relative to it were computed as $\varphi_{i}^{j}={\tilde{\varphi }}_{i}^{j}-\varphi_{3}^{3}$, where ${\tilde{\varphi }}_{i}^{j}$ are absolute phases. Phases of antennas that have a significant impact on the exposure are shown at the bottom of the Figure 4. Antennas from four central tiles $\left(\varphi_{3}^{2},\;\varphi_{2}^{3},\;\varphi_{2}^{2}\right)$ tend to have higher impact on psSAR_10g_ than those from periphery.

In all optimization runs, the antenna powers converged to their upper bound (1 mW). In fact, the optimal antenna phases guarantees, that all the antennas signals interfere constructively in the region of interest (peak-10g-cube). Therefore, increasing the antenna powers necessarily leads to the increase of exposure.

After ~1000 function evaluations the optimization terminates, reaching a flat plateau (Figure 4). The resulting exposure value is considered to be the worst-case exposure in the worst-case scenario and equals to around 21.2 mW/kg. This value is almost 50% higher than the value of the highest exposure sample observed previously and more than four times the average exposure of the ATTO-floor. It places an upper bound on the exposure of the 4-by-4 ATTO-cell array.

Figure 5 depicts the worst-case psSAR_10g_ distribution in a 2D slice, coincident with the highest exposure voxel. The voxel is located at the very edge of the right foot toe. The 10 g cube assigned to it has the volume of nearly 48 cm^3^ and only one-fifth of it is occupied with a lossy media.

**Figure 6.**
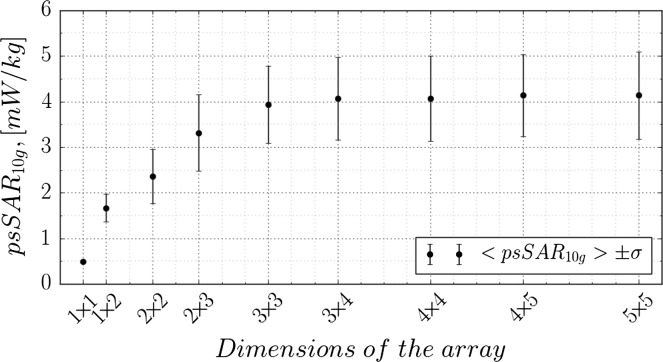
Parameters of exposure samples distributions as a function of antenna array size. Each point and error-bar represents mean value and standard deviation of exposure samples distribution.

In addition, influence of the array size on the total exposure was studied. The setup of Figure 1 was used with the antenna array extended to 5-by-5 size. The phantom position was fixed at the center of the floor with 0° rotation. After a single multi-port simulation was done, nine post-processing runs were performed and the results are shown in Figure 6. The horizontal axis indicates the size of a rectangular sub-array that was excited, gradually expanding from the central tile to the full 5-by-5 floor. Position of points and error-bars along the vertical axis indicates the average and standard deviation of 1000 random exposure samples, respectively.

The rate at which exposure grows decreases drastically after the array size becomes larger then 3-by-3 tiles. Relative exposure increase of 5-by-5 compared to 3-by-3 array is only around 2.5%. Such a small relative change of exposure justifies the use of 4-by-4 array setup with phantom movements, as in any configuration the phantom is kept enclosed inside one of four 3-by-3 sub-arrays of the initial array. At the same time, a 2-by-2 array, though covering the phantom’s footprint, is not sufficient for exposure estimation.

**Figure 7.**
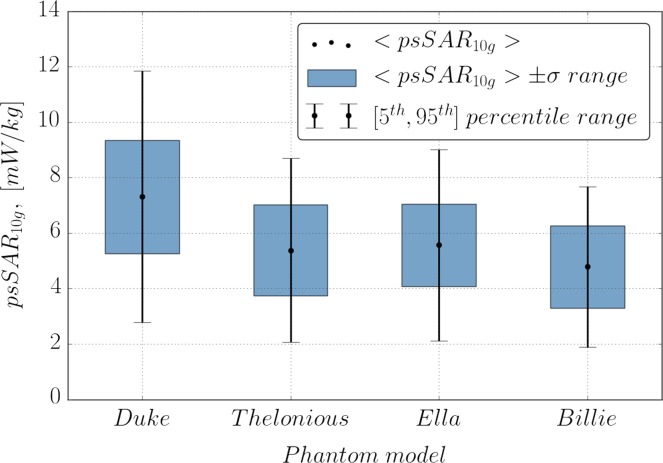
Bar-plot of psSAR_10g_ for Duke v.3.1, Thelonious v.1.0, Ella v.1.0 and Billie v.1.0 phantom models, evaluated in the worst-case configuration. Solid dots indicate mean psSAR_10g_, blue bars and caped solid lines cover $\langle {\mathrm{psSAR}}_{10g}\rangle \pm \sigma$ band and range from 5th to 95th percentile, respectively.

Finally, the effect of body morphology is investigated. Three additional simulations with different phantoms are done: Thelonious (a 6-year-old boy), Ella (an adult woman) and Billie (an 11-year-old girl). All phantoms were simulated in a worst-case configuration found for Duke (Figure 5). Figure 7 depicts the parameters of distribution for 1000 random exposure samples, generated for each phantom (including Duke). The average exposure for Thelonious, Ella and Billie phantoms is ~5.2 mW/kg, which is nearly equal to the average exposure over the whole ATTO-floor, found for Duke (4.9 mW/kg). In addition no significant variation of exposure mean among three newly simulated phantoms is present; their relative differences are 2, 6 and 9% for Thelonious, Ella and Billie phantoms, respectively. At the same time the average exposure for Duke in the same configuration (7.4 mW/kg) is nearly 30% higher. These observations suggest that the worst-case configuration found for Duke does not guarantee the worst-case for other phantom models, i.e. worst-case configuration is model-specific and possible effects of body morphology (e.g. the size of the feet) are suppressed by exposure alteration due to phantom positioning. A more accurate analysis of this matter is computationally extensive (position of each phantom should be varied) and goes out of the scope of the paper.

## CONCLUSION

In this article, the exposure of ATTO-floor technology in terms of peak spatial SAR_10g_ was estimated. We showed the significance of the effect that a multi-antenna interference has on the SAR_10g_ value and used a statistical approach to obtain the average exposure level of 4.9 mW/kg and a 95th value of 7.6 mW/kg on the ATTO-floor as well as draw a theoretical maximum for 4-by-4 floor (21.9 mW/kg). Peaks of SAR were found to occur always in feet, being well below the corresponding ICNIRP guidelines for the general public. The relation between the ATTO-floor size and SAR_10g_ it induces was established. The presented results can be used as a reference for the comparison of human exposure in wireless networks of the next generation and methods can be adapted for exposure assessment in other systems with multiple interfering sources, e.g. 5 G massive MIMO antenna arrays.
